# A Narrative Review of Toxic Heavy Metal Content of Infant and Toddler Foods and Evaluation of United States Policy

**DOI:** 10.3389/fnut.2022.919913

**Published:** 2022-06-27

**Authors:** Emily C. Bair

**Affiliations:** Division of Pediatric Gastroenterology, Michigan Medicine, University of Michigan, Ann Arbor, MI, United States

**Keywords:** heavy metals, infant foods, weaning foods, Clean Label, arsenic, lead, mercury, cadmium

## Abstract

Excessive exposure to inorganic contaminants through ingestion of foods, such as those commonly referred to as heavy metals may cause cancer and other non-cancerous adverse effects. Infants and young children are especially vulnerable to these toxic effects due to their immature development and high ’food intake/ body weight' ratio. Concerns have been raised by multiple independent studies that heavy metals have been found to be present in many foods in the infant and child food sector. Most recently, reports from the U.S. House of Representatives Subcommittee on Economic and Consumer Policy suggest subpar testing practices, lenient or absent standards, and limited oversight of food manufacturers perpetuate the presence of these contaminants in infant and toddler foods. The aim of this narrative review is to evaluate the current state of policies in the United States designed to safe-guard against excessive heavy metal exposure and to discuss what is presently known about the presence of the so-called heavy metals; arsenic, lead, mercury and cadmium found in infant and toddler foods. PubMed was used to search for studies published between 1999 and 2022 using a combination of search terms including: “heavy metal,” “contamination,” “infant,” “toddler,” and “complementary food”.

## Introduction

Concerns have been raised by multiple independent studies that inorganic contaminants, including those commonly known as ’heavy metals', have been found to be present in many foods designed for infants and young children. Most recently, reports from the U.S. House of Representatives Subcommittee on Economic and Consumer Policy suggest subpar testing practices, lenient standards, and limited oversight of some of the largest baby food manufacturers perpetuate the presence of heavy metals in infant and toddler foods (1, 2). This is a concerning public health issue as baby food manufacturers hold a special position of consumer trust.

While excessive exposure to inorganic contaminants impacts all humans; infants, toddlers, and children are especially vulnerable to their carcinogenic and non-carcinogenic effects due to their immature development and high ’food intake/ body weight' ratio. Adverse effects of inorganic contaminant exposure to infants and children may include: anemia, nephrotoxicity, developmental and reproductive toxicity, lower intelligence quotient (IQ), and neurotoxic effects (3, 4).

Multiple organizations have claimed to have detected heavy metals in varying concentrations in baby and toddler foods in the last decade and yet little has changed in the quality control or actual toxic metal content of infant and toddler foods. The National Food and Health Survey of 2017 found that nearly 30% of American adults were not confident in the safety of the US food supply with specific concerns for the presence of carcinogens increasing between the 2016 and 2017 surveys (5). Non-governmental organizations, like The Clean Label Project, have taken independent steps toward identifying brands and products committed to reducing exposure to inorganic contaminants like heavy metals.

## Methods

This narrative review discusses what is presently known regarding the presence of heavy metals in infant and toddler foods. It additionally evaluates the current state of national policy in the United States dedicated to the evaluation and mitigation of heavy metal contamination of infant and toddler foods. PubMed was used to search for studies published between January 1999 and April 2022 using the keywords: “heavy metal,” “contamination,” “infant,” “toddler”, and “complementary food”. To be included, the manuscript had to have evaluated the presence of either arsenic, lead, mercury, cadmium or a combination thereof in food products designed for infants and/ or toddlers (eg., Formula, purees, cereals). Studies evaluating the presence of heavy metals in breast milk and fetal exposure were not included. Studies were selected based on their relevance to a discussion about United States food policy. Only articles available in English were included.

## Heavy Metals: Arsenic, Lead, Mercury and Cadmium

The term ’heavy metal' is widely used in the scientific community without a standardized definition. According to the FDA, the metals mercury and lead and metalloid arsenic are defined as “heavy metals.” In this article, inorganic arsenic, lead, mercury and cadmium are referred to collectively as heavy metals as they have been collectively analyzed together in previous studies.

Arsenic, in its inorganic form, has been ranked by the Department of Health and Human Services' Agency for Toxic Substances and Disease Registry (ATSDR) as the number one most significant substance present in the environment to pose a threat to human health (6). Health risks known to be associated with arsenic exposure include impacts to multiple organ systems - pulmonary, gastrointestinal, hematological, hepatic, renal, immunologic, and neurological. The most severe effects being damage to the central nervous system and cognitive development in children (7).

Lead is second on ATSDR's list of harmful environmental substances (6). Similar to inorganic arsenic, lead exposure has been associated with a number of poor health outcomes including decreased cognitive performance, behavioral problems, delayed puberty and stunted postnatal growth. Most concerning is that the cognitive effects that lead exposure in early childhood contributes to appear to be permanent. In one follow-up prospective study, individuals who had previously experienced lead-associated developmental delays in childhood continued to show persistent cognitive deficits into adulthood (8).

Mercury (or methylmercury) is third on ATSDR's list of harmful environmental substances (6). Unlike arsenic and lead, studies on mercury's effect on childhood development have largely focused on the impact it may have via maternal exposure during pregnancy. In these instances, prenatal mercury exposure has consistently been found to be associated with poor neurodevelopment and lowered IQ (9).

Cadmium is the fourth heavy metal on ATSDR's list of harmful environmental substances, but it ranks at number seven on ATSDR's list overall (6). Following suit with arsenic, lead, and mercury, cadmium exposure in infancy and young childhood has been found to negatively affect Full Scale IQ scores (10). Cadmium exposure has also been found to be linked to increased incidence of Attention-Deficit/ Hyperactivity Disorder (ADHD) (11).

## Toxicity of Heavy Metals

Chronic oral exposure to heavy metals has been associated with cancer and other adverse non-cancer health effects as described above. Exposure to heavy metals during acute developmental stages can contribute to “untreatable and frequently permanent” neurological damage resulting in “reduced intelligence” or “disruption of behavior” (3). In a study on the impact of contributions of environmental chemicals on lost IQ points in U.S. children it was determined that exposure to environmental chemicals, such as lead, have been associated with the loss of 40,131,518 IQ points in roughly 25.5 million children (or about 1.57 lost IQ points per child). This is more than the total IQ losses found to be associated with preterm birth (34,031,025), brain tumors (37,288), and traumatic brain injury (5,827,300) combined (12).

Heavy metals have been found to be highly interactive with biological systems. Human cells have numerous ligands for binding to chemical elements, including essential and non-essential elements. Metals have a particular affinity for binding sulfhydryl groups of proteins and their interactions with these loci can inhibit the production of more than 200 enzymes in the human body. Heavy metals may also interact with cells through mimicry. Through mimicry these non-essential elements can attach to physiological sites normally reserved for essential elements thereby disrupting normal biochemical and/or physiological functions. Additionally, heavy metals may act as catalysts for oxidoreductive reactions with oxygen or other endogenous oxidants to produce oxidative modifications to proteins and DNA. This is likely the pathway by which heavy metals contribute to carcinogenicity (13).

The pediatric population is more vulnerable to toxic elements due to their high intestinal absorption capabilities, low effective excretion and high ’food intake/ body weight' ratio (13). The toxicity of heavy metals in children depends on the exposure frequency to a specific metal. Children are estimated to consume three-times as much food as adults when compared to their body mass (14). It can therefore be surmised that their risk for accumulation of toxic elements from their dietary intake is at minimum triple that of adults. This is additionally confounded by the fact that infant and early childhood foods are routinely made with products that have been shown to contain higher levels of environmental toxins.

## Occurrence of Heavy Metals

Heavy metals are contributed to the environment in ground water, forest fires and volcanic ash, natural erosion, factory and power plant emissions. Consequently, the raw materials used to prepare infant and toddler processed foods (milk, fruits, vegetables, cereals, etc.) have the potential to contain several chemical elements with toxic properties. The inorganic contaminant load of a raw ingredient may be impacted by anthropogenic activities and natural phenomena. Metals have a particular ability to accumulate in foods as they are not subject to traditional biodegradation processes. Instead, they are readily absorbed by sediments and biomagnified through the food chain. Additional processing of these raw materials into food products, such as the addition of inorganic salts, may increase the risk for toxic heavy metal exposure (13).

Grains, especially rice, are particularly impacted by environmental heavy metal contamination in water and soil. In heavily contaminated regions, rice has been found to have a 10-fold elevated level of inorganic arsenic present as compared to other foods (15). Rice contains much higher levels of arsenic due to being the only major cereal crop grown under flooded conditions. Additionally, rice and sweet potatoes have been identified as the most common ingredients to contain lead. Processed “kid's meals” and grain-containing foods such as rice, quinoa, wheat, and oats have been found to frequently contain an abundance of cadmium (16).

Rice is widely used as an ingredient in weaning products in rice cereals. A child's intake of rice products dramatically increases after weaning from breast milk/ formula feedings and this contributes to dramatic increases in the potential exposure to heavy metals in baby foods. In fact, one study found a 4.5-fold increase in inorganic arsenic and its metabolites (monomethylarsonic acid or MMAA and dimethylarsinic acid or DMAA) in urine samples from infants following weaning as compared to samples taken prior to weaning (14). Surveys of arsenic and other heavy metals in rice-based weaning/ infant and child foods have led to a growing concern for the continued utilization of rice as the main grain found in children's diets. Consequently, the removal of rice-based grain products from infant and toddler diets has been of particular interest to independent organizations like Healthy Babies Bright Futures (HBBF) and their actions have contributed to the removal of infant rice cereal from U.S. state WIC programs in Oregon, Alaska and Hawaii and made way for a wave of alternative infant cereal products to hit the market (17).

In the United Kingdom (UK), rice alternatives favored by manufacturers include maize (corn), oats, quinoa, and potato. The mycotoxin content of maize and oats however remains problematic. Studies suggest the dilution of rice with other gluten-free grains and utilization of low-arsenic rice appears to have had a compelling and positive effect on arsenic exposure in the UK markets. In one study, the median concentration of inorganic arsenic found in multigrain cereals (even those containing some rice) was 9.75 micrograms/kg a 10th below the established European Commission's (EC) threshold for arsenic in drinking water. Of note, this is substantially below the EC's new “stricter” arsenic limits of 100 micrograms/kg for infant food products (the threshold for adult foods is 200 micrograms/kg) (15).

Grains and other produce are not the only potential source of heavy metals found to be frequently incorporated into baby food products. Based on internal review documents collected by the U.S. Congressional Committee on Economic and Consumer Policy in 2021 many manufacturers use additional ingredients that test positive for heavy metals. These ingredients include items such as vitamin premixes, enzymes, and flavorings like cinnamon, cumin, and turmeric (1, 2).

## Analysis and Quantification of Heavy Metals

While experts consider cereals, produce (fruits and vegetables) and tap water to contribute the most dietary exposure to heavy metals there is very little guidance on what levels are considered safe for infants between 6 months and beyond when complimentary foods are introduced. There are few specific governmental guidelines in the United States for heavy metal contamination in foods marketed for infants and young children. The few exceptions include maximum lead and arsenic levels in certain fruit juices and inorganic arsenic in infant rice cereal. The absence of authoritative regulations and comprehensive guidance on transparent evaluation of heavy metals in food products has instigated critically important assessments of the presence of heavy metals in infant and toddler foods. Analytical techniques for assessing the presence of heavy metals in foods include spectrometric, chromatographic, and electrochemical analysis (12). These studies have been conducted at various levels of scientific rigor by governmental, non-governmental, and scientific agencies.

The Feeding Infants and Toddlers Study (FITS) of 2016 assessed the food consumption among United States infants (6 to 11.9 months) and toddlers (12 to 23.9 months). The goal was the establish the energy, nutrient contribution, and contaminant load of various food categories to the overall diet of infants and toddlers. It was based on dietary survey's including a 24-h dietary recall and feeding practices questionnaire conducted with 3,248 caregivers and was the largest cross-sectional dietary intake survey of its time in the US focused on children (18). Using data from the most recent US Food and Drug Administration's Total Diet Studies (FDA TDS) and estimations of average intake of specific foods from FITS 2016 data, Callen et al. (19) identified the presence of lead, cadmium, arsenic, and mercury in several baby foods. This study was limited by the fact that it extrapolated data from two separate surveys and did not evaluate the contaminant intake risk.

Other studies based on a selection of food samples offer additional glimpses into the presence of heavy metals in the food market but do not offer a risk assessment. For example, a total of 50 packaged foods, including some baby and toddler foods were sampled by *Consumer Reports* in 2017. All sampled products were found to contain detectable concentrations of one or more heavy metals (4). Similarly, in 2019, a non-profit known as Healthy Babies Bright Futures (HBBF) completed their first “baby food report,” an independent study on the presence of toxic heavy metals in commercial infant food products. They reported detecting at least one heavy metal in 95% of the 168 baby foods tested. They submitted a report to the public which included the top fifteen most consumed foods by children under the age of 2 years that they estimated accounted for “55% of the risk for brain development due to their high heavy metal content”. This list included apple and grape juice, oat ring cereal, macaroni and cheese, and puff snacks amongst others. HBBF called upon the FDA at this time to take immediate action and establish a proactive testing program for heavy metals like the Consumer Product Safety Commission's program for children's toys (4, 17, 20).

In November of 2021, the Congressional Subcommittee on Economic and Consumer Policy requested internal reports and tests for heavy metals from seven of the largest baby food manufacturers in the United States. These results were published in February 2021, as a part of a detailed staff report (1). Of the seven baby food manufacturers identified by the committee, only four of the companies responded to the Subcommittee's initial requests, these included Nurture, Inc. which sells Happy Family Organics and baby food under the brand name HappyBABY; Beech-Nut Nutrition Company; Hain Celestial Group, Inc. which sells baby food products under the brand name Earth's Best Organic; and Gerber. Campbell Soup Company which sells baby foods under the brand name Plum Organic; Walmart Inc. which sells baby foods under the private brand Parent's Choice; and Sprout Organic Foods Inc. refused to cooperate initially. However, following publication of the staff report in February these three companies that had originally not participated started cooperating to varying degrees. In September 2021, a second report including an evaluation of the available internal reports and tests from all seven manufacturers was released (2). As expected, the results of the committee's follow up review revealed widespread concerns for a lack of attention to toxic metal testing and relaxation of previously protective measures. Based on the subcommittee's findings accumulated over the course of these two reports it was determined that all heavy metals were found in at least a selection of products from all companies that provided testing results (1, 2). The results are summarized in Table 1.

**Table 1:** Summary of findings from U.S. congressional subcommittee on economic and consumer policy reports on heavy metal content of top seven baby food manufacturers in the United States.

	**Phase I**.				**Phase II**.		
	**Happy BABY**	**Beech-Nut**	**Earth's Best Organic**	**Gerber**	**Plum Organics**	**Parent's Choice**	**Sprout Foods, Inc**
Arsenic	>25% all products >100 ppb >50% all products >50 ppb	Products not tested, 45 ingredients >100 ppb	Products not tested, 24 ingredients >100 ppb	Products not tested, 67 batches rice flour >90 ppb	100% rice products >200 ppb	Not tested	Not tested
Lead	~8% all products >20 ppb ~19% all products >10 ppb	Products not tested, 483 ingredients >5 ppb	Products not tested, 88 ingredients >20 ppb	Limited testing	~55% all products >5 ppb	Not tested	Not tested
Mercury	56 products >2 ppb	Not tested	Not Tested	Limited testing	Not tested	Not tested	Not tested
Cadmium	65% all products >5 ppb	Products not tested, 105 ingredients >20 ppb	Products not tested, 102 ingredients >20 ppb	Products not tested, 75% carrots (ingredient) >5 ppb	~38% all products >5 ppb	Not tested	Not tested

*ppb, parts per billion*.

The Economic and Consumer Policy reports identify limitations in the testing practices currently in place for most companies. Based on company policy statements, testing practices for toxic heavy metals often only test individual ingredients instead of finished products. This can drastically underestimate the toxic heavy metal content of the consumable product and does not provide transparent information. Despite having arbitrary internal standards set, manufacturers frequently sent products exceeding their own internal standards for heavy metal content to market for sale. Hain justified their deviation from internal standards by stating their ingredient testing standards were based on “theoretical calculations” (2).

Interpretation of these studies (Economic and Consumer Policy, HBBF, and Consumer Reports) are limited due to a lack of evaluation of health risk. The reports are characterized by hazard presence (i.e., the detection of one or more heavy metals) rather than probabilistic risk assessments. While some studies have made their datasets publicly available for further evaluation [e.g., Environmental Defense Fund (EDF)] others have yet to do so (e.g., Consumer Reports). Other data sets, such as the reports collected by the H.R. Congressional committees are incomplete, containing only data on individual ingredients rather than consumable products, thereby limiting the possibility of further risk assessment. The lack of authoritative benchmarks for these food products has also limited the comparison of acceptable heavy metal concentrations. For example, the 2019 HBBF study benchmarked metal concentrations against non-authoritative limits established by advocacy groups like the EDF, American Academy of Pediatrics (AAP), Consumer Reports, as well as their own standards in lieu of absent standards from the FDA (17).

Contrary to the previously discussed studies, Parker et al. (21) evaluated the presence of and calculated exposure and risk assessments for arsenic, lead, mercury and cadmium in fruit, grain, leguminous vegetable, and root vegetable based baby foods to assess health risk of consumption of these baby food categories.

Following standard risk assessments guidelines, this study established quantification ranges, limit of detection and upper-bound exposure concentrations following a method validated by the FDA. Exposure assessment was broken into 3 age groups (birth to <1 year, 1 year to <2 years, and 2 years to <3 years) and calculations to determine average daily dose (ADD) and lifetime average daily dose (LADD) included data on mean ingredient-specific intake rates from the EPA (21).

Mass spectrometry assessment of samples revealed that arsenic was found to be present in 100% of grain and root vegetable samples, 78% of leguminous vegetable samples, and 67% of fruit samples. Lead was also found in 100% of grain samples, 88% of root vegetable samples, 33% of fruit samples, and 22% of leguminous vegetable samples. Cadmium was found in 100% of grain samples, 67% of root vegetable samples, and 33% of fruit samples. Mercury was undetected in all food samples. Non-cancer health risk was evaluated based on calculating hazard quotients (HQ) for each heavy metal, within each food group, for each age range (21).

Based on these assessments, Parker et al. (21) found human health risks (cancerous or non-cancerous) are not expected for the level of cadmium and mercury found in all sample foods. Similarly, Martins et al. (22) assessed the total mercury concentrations in infant foods available in Portugal and found that the provisional tolerable weekly intake (PWTI) in foods, other than fish and shellfish, was not exceeded. However, Parker et al. (21) did find arsenic and lead concentrations in certain product types represent potential health risks under the exposure assumptions of their assessment. They found health risks (cancer and non-cancer) associated with arsenic were driven by its presence in grain products but not fruit or vegetable products. Similarly, a risk assessment by Shibata et al. (23) concluded that median and upper bound consumption of arsenic found in rice cereal products exceeded the tolerable chronic non-cancer risk levels however, found them within an acceptable cancer risk range.

Parker et al. (21) found health risks (non-cancer) associated with lead were observed in grain, fruit, and root vegetable products. Gardener et al. (16) tested 564 foods designed for infants and toddlers for the presence of lead and cadmium and found detectable levels in 37% and 57% of samples, respectively but fewer than 7% of the total solid baby food samples collected exceeded the FDA and World Health Organization's (WHO) limits for both lead and cadmium even under high-consumption scenarios. Comparatively an analysis of Total Diet Study (TDS) data from 2014 to 2016 by Spungen (24) demonstrates that dietary lead exposure exceeded toxicity criteria when the upper bounds of data were used and cadmium exposures exceed toxicity standards across upper and lower bounds.

Akonnor and Richter (25) also evaluated the presence of lead, cadmium, mercury, and arsenic in 132 individual Stage 2 baby food products purchased from local supermarkets in the United States. Overall, only six samples were found to have one tested heavy metal measure outside of the established limits. This evaluation was limited by the fact that all products were evaluated against FDA and EU guidance limits set for specific food products such as apple juice (FDA), rice cereal (FDA), and infant formula (EU).

## Current Standards for Heavy Metals

A common denominator in the limitation of comparison of the studies outlined above is the lack of authoritative regulatory reference limits for heavy metals. The FDA has affirmed that inorganic arsenic, lead, mercury and cadmium are dangerous substances with “no established health benefit” often contributing to “illness, impairment, and in high doses, death.” According to the FDA, “even low levels of harmful metals from individual food sources” can build up in the immature biological systems of infants and children to levels of concern (26).

Currently infant food manufacturers are free to set their own internal standards for toxic heavy metal content of most ingredients and products. Based on internal evaluations, most have set those standards dangerously high and often continue to use ingredients and sell products that exceed their own set limits. Based on information obtained from Nurture (makes HappyBABY products), internal standards are used as “goal thresholds” that “are not used to make product disposition decisions and are not a precondition to product release.” Nurture even set its internal standard for arsenic in their infant rice cereal products to 115 parts per billion (ppb), 15% higher than the FDA's set standard of a maximum of 100 ppb (1, 2).

Globally a wide range of proposed and existing thresholds for heavy metal content in water and food products exist, with few pertaining specifically to infant and toddler foods. In fact, in the United States there is only one current standard in place that places limits on arsenic in infant rice cereals up to 100 ppb. Tables 2–5 summarize several proposed and existing standards for heavy metals established by the United States, European, and other international agencies.

**Table 2:** Proposed and existing inorganic arsenic standards.

**Group or agency**	**Standard**
Healthy Babies Bright Futures	No measurable amount in baby foods
Consumer Reports	3 ppb
FDA	10 ppb for bottled water 100 ppb in infant rice cereal
EPA, EU, WHO	10 ppb for drinking water
EC	100 μg/kg (for infant/ child foods) 200 μg/kg (for adult foods)

*EC, European Commission; EPA, Environmental Protection Agency; EU, European Union; FDA, Food and Drug Administration; ppb, parts per billion; WHO, World Health Organization*.

**Table 3:** Proposed and existing lead standards.

**Group or agency**	**Standard**
Environmental Defense Fund	1 ppb (especially for baby foods)
Consumer Reports	1 ppb in fruit juices
American Academy of Pediatrics (AAP)	1 ppb for water fountains in schools
FDA	5 ppb for bottled water 50 ppb for juice 100 ppb for candy
WHO	10 ppb provisional guideline
EPA	15 ppb for drinking water (action level)
EU	20 ppb for “infant formula and follow-on formula”

*EPA, Environmental Protection Agency; EU, European Union; FDA, Food and Drug Administration; ppb, parts per billion; WHO, World Health Organization*.

**Table 4:** Proposed and existing cadmium standards.

**Group or agency**	**Standard**
Healthy Babies Bright Futures	No measurable amount in baby foods
Consumer Reports	1 ppb in all fruit juices
WHO	3 ppb for drinking water
EPA/ FDA	5 ppb for drinking water
EU	5-20 ppb for infant formula

*EPA, Environmental Protection Agency; EU, European Union; FDA, Food and Drug Administration; ppb, parts per billion; WHO, World Health Organization*.

**Table 5:** Proposed and existing mercury standards.

**Group or agency**	**Standard**
Healthy Babies Bright Futures	No measurable amount in baby foods
EPA	2 ppb for drinking water

*EPA, Environmental Protection Agency; ppb, parts per billion*.

## Establishing New Action Levels for Heavy Metals

Within two weeks of publication of the aforementioned staff report from the Congressional Subcommittee on Economic and Consumer Policy the FDA issued a constituent update entitled “FDA Response to Questions About Levels of Toxic Elements in Baby Food, Following Congressional Report” which states a commitment by the FDA to “reduce exposure to toxic elements in foods to the greatest extent feasible.” Two months later the FDA announced the creation of the ’Closer to Zero Action Plan', a timeline for publishing actionable limits for arsenic, lead, mercury and cadmium particularly for infant and child foods and cereals (27).

The FDA establishes action levels following a four-step approach (1) evaluation of scientific data, (2) establishment of interim reference levels (IRLs) and proposed action levels, (3) consultation with stakeholders to discuss achievability and feasibility and (4) finalization of action levels. Once action levels are finalized and made available to the appropriate industries, the FDA will establish a timeline with manufacturers for meeting progress toward achieving these action levels (27, 28). Tables 6–8 outline the latest proposed timelines for establishing action levels for heavy metals.

**Table 6:** Phase 1 FDA's Closer to Zero Action Plan: April 2021 - April 2022.

**Stage of improvement**	**Toxic metal**	**Action**
Evaluate	Arsenic	Gather data to work toward proposal of IRL
Propose	Lead	Draft action levels
Consult	Lead	Engage with stakeholders
Monitoring/ compliance	All	Sampling, education on best practices

*IRL, interim reference level; FDA, Food and Drug Administration*.

**Table 7:** Phase 2 FDA's Closer to Zero Action Plan: April 2022 - April 2024.

**Stage of improvement**	**Toxic metal**	**Action**
Evaluate	Cadmium Mercury	Gather data to work toward proposal of IRL
Propose	Arsenic	Draft action levels
Consult	Arsenic	Engage with stakeholders
Finalize	Lead	Finalize action levels
Monitoring/ compliance	All	Sampling, education on best practices

*IRL, interim reference level; FDA, Food and Drug Administration*.

**Table 8:** Phase 3 FDA's Closer to Zero Action Plan: April 2024 – Beyond.

**Stage of improvement**	**Toxic metal**	**Action**
Evaluate	Lead	Gather NEW data to work toward further reduction in action level
Propose	Cadmium Mercury	Draft action levels
Consult	Cadmium Mercury	Engage with stakeholders
Finalize	Arsenic	Finalize action levels
Monitoring/ compliance	All	Sampling, education on best practices

*FDA, Food and Drug Administration*.

The congressional subcommittee has since recommended acceleration of the above proposed timeline set forth by the FDA. They have been joined in their pursuit by a coalition of 23 attorney generals led by New York Attorney General Letitia James. The attorney generals have petitioned the FDA to “move expeditiously to set limits for arsenic, lead, cadmium, and mercury in baby foods.” They propose these actions occur no later than April 2022 (28). Additionally, in light of the initial report from the Economic and Consumer Policy Subcommittee, Rep. Raja Krishnamoorthi (Illinois) sponsored H.R. 2229, or The Baby Food Safety Act, which was introduced to the House Committee on Energy and Commerce on March 26, 2021. The Baby Food Safety Act outlines the establishment of max levels of certain toxic elements (including heavy metals inorganic arsenic, lead, mercury and cadmium) allowable in infant and toddler foods designed for children up to 36 months-of-age (28). See Table 9 for more details about the recommended initial action levels by The Baby Food Safety Act (28).

**Table 9:** Recommended initial action levels from the Baby Food Safety Act (H.R. 2229).

**Toxic heavy metal**	**Initial action level**
Inorganic arsenic	10 ppb for infant and toddler foods 15 ppb for infant and toddler cereals
Lead	5 ppb for infant and toddler foods 10 ppb for infant and toddler cereals
Mercury	2 ppb for infant and toddler foods/ cereals
Cadmium	5 ppb for infant and toddler foods 10 ppb for infant and toddler cereals

*ppb, parts per billion*.

The bill also calls on the FDA to periodically review and further lower these established levels as needed based on the review of relevant health and dietary data. It also requires facilities that manufacture, process, pack or hold infant and toddler foods to have certain controls and plans to ensure their food products comply with these limits on toxic elements and this data should be made publicly available. The Baby Food Safety Act expands the FDA's authority to require a recall of adulterated and/ or misbranded foods that exceed these limits on toxic elements. It outlines a plan for the CDC to carry out a public awareness campaign outlining the risks of toxic elements in infant and toddler foods. They also encourage the FDA to commission research on agricultural practices that may minimize levels of toxic heavy metals in crops (28).

## Beyond Action Limits: Organic and Clean Label Certification

There are clearly challenges faced by the FDA to moving forward with the previously outlined plans for establishing new action limits and this has driven consumers to seek safe options for infant and toddler foods elsewhere meanwhile. Some have turned to the organic market which is growing in baby food products. Organic food sales reached nearly $56 billion in 2021 based on the U.S. Organic Industry Survey by the Organic Trade Association. This study also revealed that “baby food” and “foods targeted at kids” ranked as top categories when respondents were asked which foods are most important to purchase organic (29).

While certified organic foods have been shown to have fewer pesticides than non-organic foods there is limited clear scientific evidence to support that organic practices also contribute to lower heavy metal content of foods. In fact just one study was identified which investigated the cadmium content of organic baby foods in Spain. However, this study did find the presence of cadmium to be considerably lower in organic baby food samples when compared to previous study results evaluating the bioavailability of cadmium reported in infant formulas and weaning foods not categorized as organic (30, 31). This one study suggests that the use of organic ingredients (especially those at highest risk for heavy metal contamination) could be one of the necessary commitments to reduce the presence of heavy metals in the pediatric diet, however further research is necessary to evaluate the presence of heavy metals in organic products.

Additionally, independent groups like the Clean Label Project (CLP) have committed to providing consumers with cleaner, more transparent product label information for common household products including some infant and toddler foods and beverages. The Clean Label Project is a non-profit organization that sets rigorous standards for ingredient disclosure and transparent food labeling in products that seek their approval through a Clean Label Project Purity Award (32). Hundreds of products have achieved CLP certification which involves product sampling, testing at a FDA-registered and compliant laboratory, benchmarking against other consumer goods in their category and commitment to ongoing compliance.

In addition to evaluations for other chemical contaminants the CLP analysis specifically assesses for the presence of heavy metals arsenic, lead, mercury, and cadmium. Maximum tolerance limits used by CLP for heavy metals are substantially below the available FDA guidelines and inspired mainly by the California Proposition 65 Safe Harbor recommendations (33). Current tolerance limits used by CLP are described in Table 10 (32). Less than two dozen brands of infant formulas and baby and toddler foods have been able to make this commitment and achieve this distinction. Among these companies include several infant and toddler food manufacturers such as Cerebelly, Fresh Bellies, Little Spoon, Once Upon a Farm, Serenity Kids, and Yumi and infant and toddler formula companies including Bobbie and Else Nutrition (32).

**Table 10:** Maximum tolerance limits set by the Clean Label Project for heavy metal analytes.

**Analyte**	**Maximum tolerance limit (μg/day)**	**Source/inspiration**
Arsenic	0.06 (inhalation) 10 (non-inhalation)	EPA arsenic in drinking water standard
Lead	0.5	California proposition 65
Mercury	0.3	California proposition 65
Cadmium	0.05 (inhalation) 4.1 (non-inhalation)	California proposition 65

*EPA, Environmental Protection Agency*.

## Discussion

Overall, this evaluation of current US policy and presence of heavy metals in infant and toddler foods indicates that there remain large gaps in understanding at the public level and inconsistent assessment protocols at the governmental and scientific level. Future studies evaluating the presence of heavy metals in infant foods should strive to follow reproducible risk assessment methods rather than hazard presence-based models at least until authoritative action limits can be set for these particular food items for threshold comparisons.

Continued conversation and evaluation to help characterize and methods to mitigate heavy metal contamination risks of foods designed for infants and young children provides transparency to consumers. It can be challenging to communicate risk without creating undue concern and/or unnecessary removal of foods from a child's diet. Calls to action and a plan for the establishment of new FDA action limits have been set but not at a pace that many independent organizations (e.g., CLP or HBBF) or American consumers find reasonable. The As Low as Reasonably Achievable (ALARA) Principle offers an interim approach to establishing expectations between producers and consumers until acceptable safe levels can be identified.

Recommendations from the U.S. House of Representatives Subcommittee on Economic and Consumer Policy following their 2021 reports include (1) baby food manufacturers should be required by the FDA to test their finished products for toxic heavy metals, not just their raw ingredients, (2) manufacturers should be required by the FDA to report toxic levels of heavy metals on food labels, (3) manufacturers should voluntarily phase out ingredients and products found to have high toxic heavy metal content, and (4) the FDA should set clear maximum levels or inorganic arsenic, lead, cadmium and mercury permitted in baby foods. In the mean time, independent groups like The Clean Label Project offer consumers increased transparency about the heavy metal content of certified products including some infant and toddler foods.

## Author Contributions

The author confirms being the sole contributor of this work and has approved it for publication.

## Conflict of Interest

The sole author is an independent consultant for Else Nutrition. Else Nutrition, nor any of its employees influenced the collection or interpretation of the resources included in this manuscript.

## Publisher's Note

All claims expressed in this article are solely those of the authors and do not necessarily represent those of their affiliated organizations, or those of the publisher, the editors and the reviewers. Any product that may be evaluated in this article, or claim that may be made by its manufacturer, is not guaranteed or endorsed by the publisher.
